# Co-infections and Reactivation of some Herpesviruses (HHV) and Measles Virus (MeV) in Egyptian Cancer Patients infected with Severe Acute Respiratory Syndrome Coronavirus 2 (SARS-CoV-2)

**DOI:** 10.1186/s43046-025-00275-1

**Published:** 2025-04-11

**Authors:** Ahmed M. Mayla, Waleed S. Mohamed, Abdel-Rahman N. Zekri, Nora A. Gouda, Mai M. Lotfy, Mohamed G. Seadawy, Mohamed Abdel-Salam Elgohary, Zeinab F. Abdallah

**Affiliations:** 1 Cancer Biology Department, Virology and Immunology Unit, National Cancer Institute, Cairo University, Cairo, Egypt; 2 Cancer Epidemiology and Biostatistics Department, National Cancer Institute, Cairo University, Cairo, Egypt; 3 Biodefense Center for Infectious and Emerging Diseases, Ministry of Defense, Cairo, Egypt; 4 Egypt Center for Research and Regenerative Medicine, ECRRM, Cairo, Egypt

**Keywords:** Cancer, COVID-19, SARS-CoV-2, Measles Virus (MeV), Human Herpesviruses (HHVs), Viral reactivation

## Abstract

**Background:**

Coinfections and reactivation of persistent or latent viral infections such as herpesviruses (HHV) and/or measles virus (MeV) have been reported among COVID-19 patients. However, there is limited information regarding cancer patients who experienced severe acute respiratory syndrome corona virus-2 (SARS-CoV-2). The primary purpose of this study was to investigate the interplay between SARS-CoV-2, HHV and MeV in cancer patients, aiming to provide insights into the pathophysiology of these infections and to enhance the patients’ health outcomes.

**Methods:**

A prospective observational study was conducted on 4 groups (*n* = 147): newly diagnosed cancer patients infected with SARS-CoV-2 (*n* = 37), newly diagnosed cancer patients non-infected with SARS-CoV-2 (*n* = 13), apparently normal individuals infected with SARS-CoV-2 (*n* = 82) and finally a normal control group (*n* = 15). All samples were tested for SARS-CoV-2 infection using the real-rime quantitative reverse transcription polymerase chain reaction (qRT-PCR). Antibody responses were analyzed using indirect enzyme-linked immunosorbent assay (ELISA), and antibody levels were compared between patients and controls. Potential re-activation was investigated using fourfold (i.e. 400%) rise model criterion.

**Results:**

In all positive cases of SARS-CoV-2, recent infections or re-infection of herpes simplex viruses 1 and 2 (HSV1/2 or HHV1-2) were found to be significantly increased approximately three-fold higher in COVID-19 patients (*p* = 0.007) identified via pooled HSV1/2 IgM levels in plasma. Furthermore, reactivation of HSV1/2 was 29.7% in cancer/COVID-19 patients (*n* = 37) versus 0.0% of normal/COVID-19 group (*n* = 22) (*p* = 0.008). Likewise, Epstein-Barr Nuclear Antigen-1 (EBNA-1) IgG levels showed a ≥ fourfold increase in 20% (*p* = 0.034) of cancer patients (*n* = 50) versus 4.9% of controls (*n* = 41) for reactivation of Epstein-Barr virus (EBV or HHV-4). Obviously, MeV IgG levels increased up to 78.0% in cancer patients (*n* = 50) versus 17.5% in non-cancerous group (*n* = 40, *p* < 0.001). Reactivation of MeV in cancer and COVID-19 patients was 43.2% versus 30.8% cancer non-COVID-19 group, 3.3% normal COVID-19, and 0.0% in healthy volunteers (*p* < 0.001).

**Conclusion:**

Cancer patients infected with SARS-CoV-2 were at increased risk of HHV and MeV co-infection and reactivation.

## Background

The emergence of global pandemic pneumonia outbreak in December 2019, was linked to the Huanan Seafood Wholesale Market, Wuhan, China [1]. The World Health Organization (WHO) has identified the outbreak’s causative agent as SARS-CoV-2, one of seven known human infecting coronaviruses (HCoVs) that emerged from an animal reservoir [2]. Up to date, there are 54 officially recognized coronaviruses’ (CoV) species within the *Coronaviridae* family [3]. SARS-CoV-2 causes the disease named COVID-19 [4].

The disease can result in mild, moderate or severe and critical illness, potentially leading to severe respiratory distress syndrome that requires immediate hospitalization and, in some cases, mechanical ventilation which may ultimately result in death [5]. Additional symptoms may be experienced by some patients, including cutaneous lesions and neurological disorders, alongside respiratory symptoms [6, 7]. Moreover, it can lead to life-threatening pneumoniae; particularly in immunocompromised patients, such as active cancer [8–10].

Viral reactivation or co-infection with COVID-19 has been associated with worse clinical outcomes in critically ill patients. Viral reactivation involves both lytic and latent phases. Viruses may alternate between these latent and lytic cycles, and the transitions of a latent virus into the lytic phase is referred to as reactivation [11–13]. Measles, HSV-1 and HSV-2 have utilized viral persistence strategies to persist in an immune-privileged site, the central nervous system (CNS), and over time after reactivation cause rare disease in the CNS or distant sites [14].

There are eight human herpes viruses (HHV) [15]. Throughout the COVID-19 pandemic, there were reports of rise in HHV co-existence with SARS-CoV-2 in individuals with COVID-19. This increase was linked to the mechanisms employed by SARS-CoV-2 for replication, immune modulation and lymphocytopenia as well as potential reactions to certain medications like steroids and immunomodulatory drugs. As a result, SARS-CoV-2 may assume another role as an unforeseen contributor to the lytic reactivation of HHV [16–21]. In individuals suffering from loss of immunosurveillance particularly those with cancer, HHV can lead to severe diseases [22, 23]. Consequently, it is expected that these co-infections may lead to more severe complications than those experienced by individuals without co-infections or those who are cancer-free [24].

It has been proposed that the infection with SARS-CoV-2 could trigger the reactivation of HSV, potentially leading to HSV-related diseases in patients with COVID-19 [25]. Several studies have reported the presence of anti-HSV IgM in the serum samples of COVID-19 patients who exhibited mucosal and cutaneous manifestations, suggesting that this may be related to the treatment for COVID-19 [26]. COVID-19 accompanied by HSV reactivation cases have been documented, with manifestations ranging from mild conditions, such as HSV lesions on the lips, to more severe complications, including acute liver failure due to HSV-1 and cranial polyneuropathy [20]. Added to, clinical manifestations of HSV reactivation in cancer patients often include localized infections like herpes simplex stomatitis or more severe complications such as herpes simplex encephalitis (HSE) and disseminated disease, which can lead to significant morbidity and mortality [27]. In comparison to prior HSV reactivations unrelated to COVID-19, the COVID-19-related episodes were more severe in most of cases [28].

COVID-19 patients who experienced reactivation of herpes viruses, including HSV and EBV, were found to have prolonged hospital stays compared to those without any reactivated herpesviruses [20, 29]. It was proposed that EBV reactivation in COVID-19 patients may play a significant role in the development of COVID syndrome [18, 30, 31]. Moreover, in the co-occurrence of EBV and SARS-CoV-2, SARS-CoV-2 may facilitate EBV's oncogenic potential by disrupting the immunosurveillance and inducing a state of lymphocytopenia that may lead to a rise in mortality rate [32]. Thus, these cases have been administered more immuno-supportive therapies compared to SARS-CoV-2 patients without EBV co-infection [18].

Measles is a highly contagious viral disease caused by the morbillivirus, primarily affecting children but capable of infecting individuals of all ages. With the exception of the underdeveloped regions (https://www.who.int/news-room/fact-sheets/detail/measles; [33], the introduction and widespread administration of the measles vaccine have significantly reduced the incidence of the disease (https://www.cdc.gov/measles/about/history.html). However, in recent years there has been a devastating wave of measles-related deaths that occurred in 2018 and 2019 offers strong proof that the disease cannot be allowed to “mark time” throughout the world. Particularly in many susceptible nations, the COVID-19 epidemic has left a serious immunity-gap legacy that necessitates immediate action to avert an impending measles disaster [34, 35] (https://www.mayoclinic.org/diseases-conditions/measles/symptoms-causes/syc-20374857). Despite the recent outbreaks of measles, there have been few studies examining the susceptibility of cancer patients to the virus, even though these individuals are at a significantly higher risk for complications related to infections such as viral pneumonia [36].

Given the early nature of this study and based on the foregoing, the purpose of this study is to investigate the possibility of co-infection and reactivation of some respiratory viruses that cause persistent viral infection, such as measles virus, as well as some herpes viruses which can also cause respiratory symptoms whose link to COVID-19 infection has been established in previous research [34, 37, 38].

## Results

### Demographics

Demographic data shown in (Table 1) comparing patients with and without cancer and COVID-19. The mean age of patients varies significantly across groups, with those having COVID-19 but no cancer being the oldest (50.20 years) and those without either condition being the youngest (23.40 years), with a significant *p*-value of 0.041. The presence of cancer and COVID-19 among patients shows no significant differences, with *p*-values of 0.123 and 0.182, respectively.

**Table 1:** Clinical characteristics for all studied groups

Parameter	Cancer/COVID (*N* = 37)	Cancer/No COVID (*N* = 13)	Noncancerous/COVID (*N* = 82)	Control (*N* = 15)
**Demographics**				
** Age** (mean ± sd)	46.92 ± 16.99	59.45 ± 12.88	23.56 ± 6.03	22.4 ± 1.9
** Male**	17 (45.9%)	7 (53.8%)	82 (100%)	15 (100%)
** Female**	20 (54.1%)	6 (46.2%)	0	0
**Clinical symptoms**				
** Fever**	-	-	32 (39.02%)	2 (13.33%)
** Cough**	9 (24.32%)	28 (75.68%)	39 (47.56%)	8 (53.33%)
** Fatigue and headache**	-	-	44 (53.67%)	6 (40%)
** GIT Symptoms**	1 (2.70%)	0	12 (14.63%)	0
** CT GGO**	0	0	6 (7.32%)	0
**Laboratory results**				
** Hb (g/dL)**	13.30 (5.20–16.20)	11.80 (5.20–15.80)	13.90 (9.80–16.80)	14.00 (12.60–15.60)
	***p* = 0.005**			
** TLC/µL**	6.93 (1.21–30.37)	9.41 (4.10–143.0)	6.60 (2.30–17.39)	6.50 (2.90–16.89)
	*p* = 0.071			
** Lymphocytes/µL**	34.00 (13.30–88.10)	22.45 (14.40–98.70)	28.30 (4.20–70.20)	25.90 (4.40–68.40)
	*p* = 0.499			
** PLT/µL**	219.0 (23.0–465.0)	283.00 (7.0–623.0)	187.00 (90.0–320.0)	206.00 (75.0–294.0)
	***p* = 0.007**			
** Neutrophils**	55.70 (0.40–79.0)	63.25 (1.24–78.90)	63.70 (22.30–88.20)	66.70 (25.50–90.80)
	*p* = 0.082			
** AST(IU/L)**	18.00 (8.0–65.0)	12.00 (6.0–47.0)	16.00 (8.0–179.0)	15.00 (11.0–29.0)
	*p* = 0.094			
** ALT (IU/L)**	**27.00** (10.0–112.0)	18.00 (12.0–53.0)	19.00 (10.0- 48.0)	17.00 (13.0–47.0)
	***p* = 0.004**			
** Serum creatinine (mg/dL)**	0.80 (0.50–1.90)	0.80 (0.60–1.20)	**1.10** (0.70–102.0)	0.90 (0.80–1.40)
	***p* = 0.001**			
** Blood Urea (mg/dL)**	**28.00** (14.0–98.0)	25.00 (11.0–39.0)	**30.00** (12.0–81.0)	20.00 (15.0–31.0)
	***p* = 0.009**			

### Hematological and biochemical parameters

Regarding hematological and biochemical parameters: hemoglobin (Hb) levels are notably higher in normal COVID-19, and control compared to malignant groups (*p* = 0.005). Platelet counts also show significant variation, with cancer non-COVID-19 group having the highest count and normal COVID-19 group the lowest (*p* = 0.007). ALT levels are significantly lower in all groups compared to cancer COVID-19 group (*p* = 0.004). Serum creatinine levels are significantly higher in normal COVID-19 group (*p* < 0.001), while blood urea levels are significantly different across the groups, with normal COVID-19 group having the highest and control group the lowest (*p* = 0.009). Other parameters like TLC, lymphocytes, neutrophils, and AST do not show significant differences between the groups (Table 1).

Correlations for the data in (Table 2) compares various blood parameters across groups defined by their SARS-CoV-2 qRT-PCR and IgG status, as well as MeV IgG status (Table 3). Hb levels do not show significant differences across the groups. Platelet counts (PLT) show a significant difference when comparing MeV IgG negative and positive groups (*p* = 0.026). Neutrophil counts are not significantly different across the groups. AST levels show significant differences when comparing SARS-CoV-2 IgG negative and positive groups (*p* = 0.042) and MeV IgG negative and positive groups (*p* = 0.010). ALT levels are significantly different in the MeV IgG groups (*p* = 0.025). Serum creatinine levels show significant differences across all comparisons, with *p*-values ranging from 0.011 to 0.035. Blood urea levels are significantly different when comparing SARS-CoV-2 qRT-PCR tested negative and positive groups (*p* = 0.010).

**Table 2:** Correlation between SARS-CoV-2 qRT-PCR results, IgG and MeV IgG versus laboratory investigations

Parameter	Negative qRT-PCR	Positive qRT-PCR	SARS-CoV-2 IgG Negative	SARS-CoV-2 IgG Positive	MeV IgG Negative	MeV IgG Positive
**Hb (g/dL)**	13.50 (5.20, 15.80)	13.90 (5.20, 16.80)	13.40 (5.20, 15.90)	13.80 (5.20, 16.20)	13.85 (5.20, 16.20)	13.55 (6.20, 15.80)
	*p* = 0.100		*p* = 0.400		*p* = 0.226	
**TLC/µL**	7.20 (2.80, 143.00)	6.90 (1.21, 30.37)	6.73 (1.21, 17.54)	7.10 (2.30, 143.00)	6.95 (1.21, 30.37)	7.02 (2.90, 143.00)
	*p* = 0.219		*p* = 0.204		*p* = 0.735	
**Lymphocytes/µL**	22.75 (4.40, 98.70)	31.00 (4.20, 88.10)	31.10 (13.50, 70.20)	32.00 (4.40, 98.70)	31.10 (4.40, 88.10)	33.00 (11.70, 98.70)
	*p* = 0.064		*p* = 0.645		*p* = 0.828	
**PLT/µL**	203.00 (7.00, 623.00)	197.00 (23.00, 465.00)	203.00, (7.00, 465.00)	210.00 (23.00, 623.00)	195.50 (7.00, 506.00)	234.00 (38.00, 623.00)
	*p* = 0.416		*p* = 0.422		***p* = 0.026**	
**Neutrophils**	68.25 (1.24, 90.80)	58.60 (0.40, 88.20)	58.35 (5.20, 70.00)	58.00 (0.40, 90.80)	63.10 (0.40, 90.80)	58.05 (1.24, 80.00)
	*p* = 0.061		*p* = 0.426		*p* = 0.484	
**AST (IU/L)**	19.00 (12.00, 53.00)	21.00 (10.00, 112.00)	25.50 (11.00, 53.00)	19.00 (10.00, 112.00)	18.00 (11.00, 47.00)	25.50 (10.00, 112.00)
	*p* = 0.436		***p* = 0.042**		***p* = 0.010**	
**ALT (IU/L)**	14.00 (6.00, 47.00)	17.00 (8.00, 179.00)	17.50 (8.00, 65.00)	14.00 (6.00, 57.00)	13.00 (8.00, 34.00)	17.00 (6.00, 65.00)
	***p* = **0.091		*p* = 0.228		***p* = 0.025**	
**Serum creatinine (mg/dL)**	0.90 (0.60, 1.30)	1.00 (0.50, 102.00)	0.80 (0.60, 1.20)	0.90 (0.50, 1.90)	1.00 (0.70, 1.90)	0.80 (0.50, 1.40)
	***p* = 0.035**		***p* = 0.025**		***p* = 0.011**	
**Blood Urea (mg/dL)**	24.00 (11.00, 44.00)	29.00 (12.00, 98.00)	28.00 (15.00, 40.00)	26.00 (11.00, 98.00)	25.00 (12.00, 98.00)	26.50 (11.00, 44.00)
	***p* = 0.010**		*p* = 0.730		***p* = **0.976

**Table 3:** Correlation between MeV IgG versus laboratory investigations

Parameter	r	*p-*value
Hb (g/dL)	-0.134	0.232
TLC/µL	0.063	0.572
Lymphocytes/µL	-0.035	0.757
PLT/µL	0.226	**0.042**
Neutrophils	-0.089	0.431
AST (IU/L)	0.392	** < 0.001**
ALT (IU/L)	0.374	**0.001**
Serum creatinine (mg/dL)	-0.246	**0.029**
Blood Urea (mg/dL)	0.056	0.625

### SARS-CoV-2 qRT-PCR, IgG and IgM

**Fig. 1:**
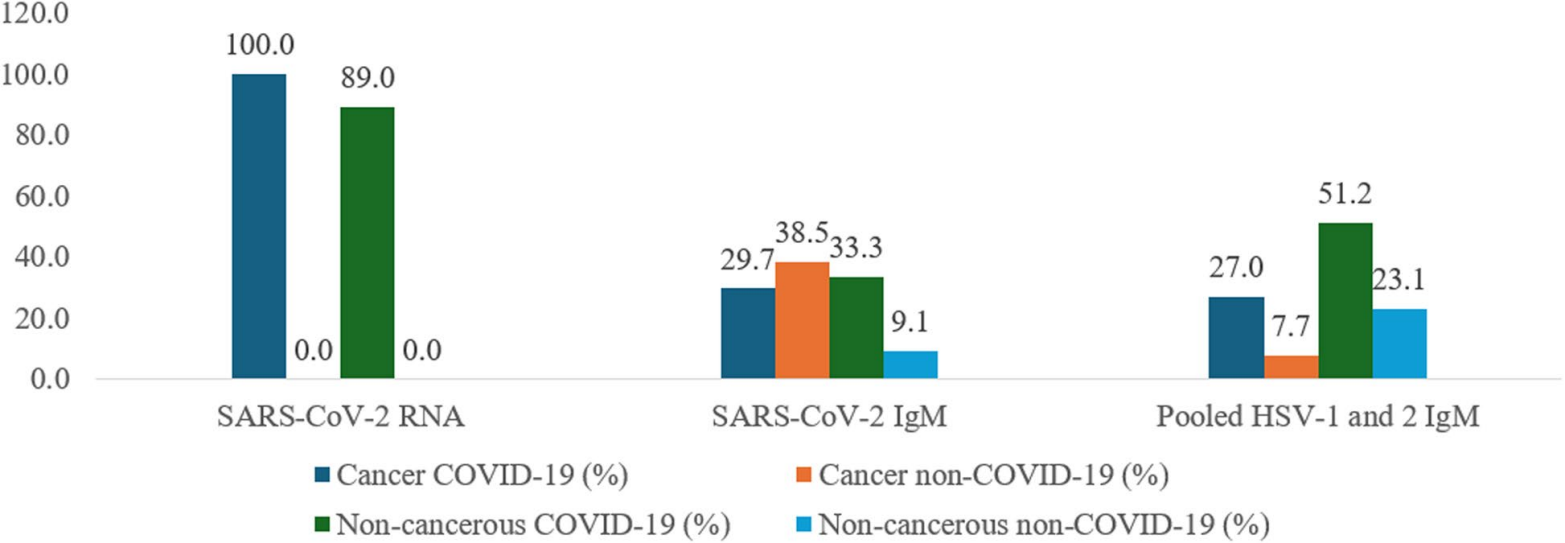
Recent infections, IgM titers, and viral SARS-CoV-2 genome in all study groups versus each other’s

Using real-time qRT-PCR to quantitatively analyze and estimate the viral loads (SARS-CoV-2 RNA), it turned out that 100.0% of the cases in the cancer COVID-19 group and 89.0% of the cases in the normal COVID-19 group were positive, respectively, while the cases in the non-COVID-19 groups were both negative (*p* = 0.001) (Fig. 1).

**Fig. 2:**
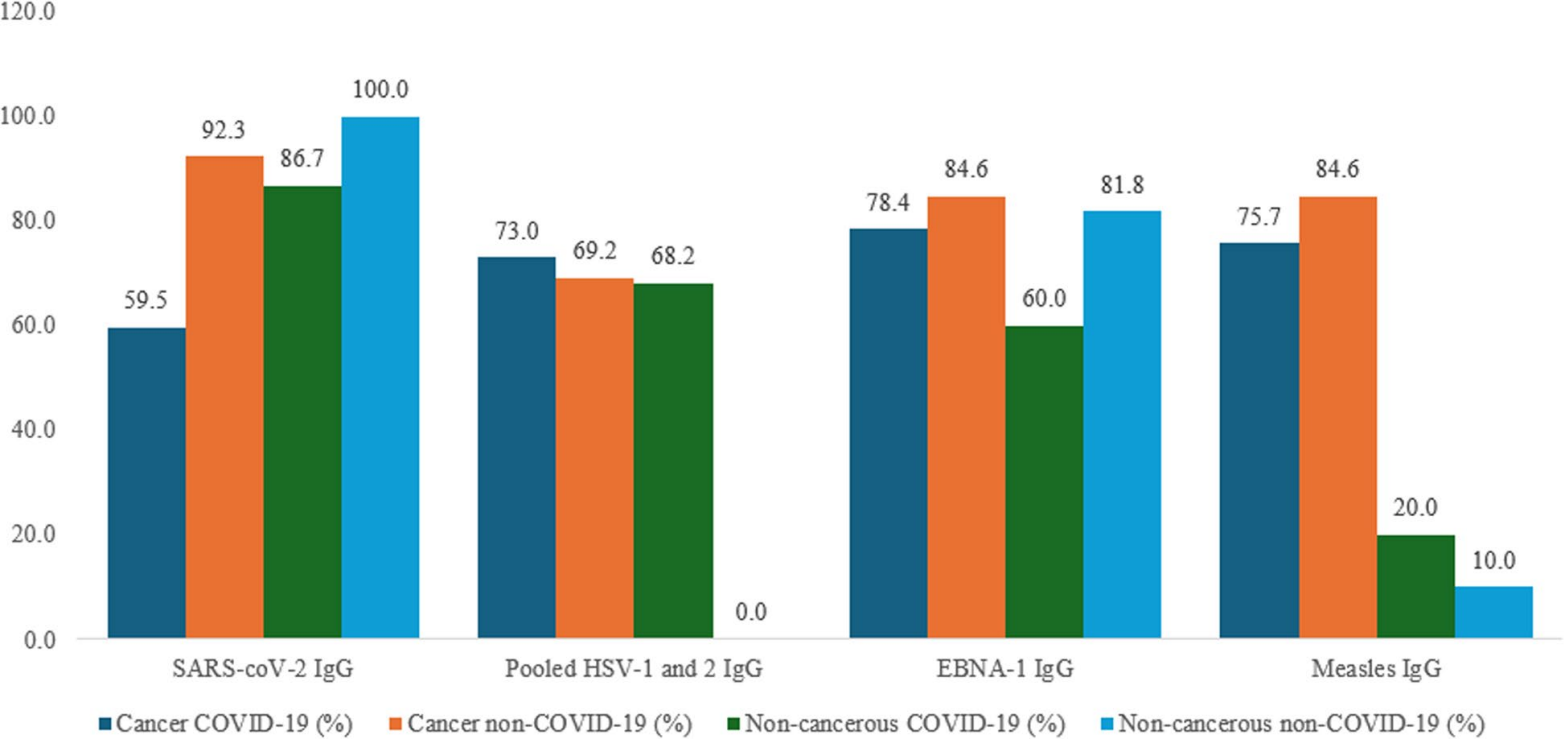
IgG titers in all study groups versus each other’s

To investigate potential viral reactivation of various immunoglobulins formed against active viral infections during infection with COVID-19, we examined the immunoglobulins for antibodies to the viruses under investigation. When examining the immunoglobulins SARS-CoV-2 IgG, 100.0% of the cases in the control group were positive, while 86.7% of the cases in the normal COVID-19 group were positive, compared to 92.3% in the cancer non-COVID-19 group. Merely 59.5% of positive cases were found in the cancer COVID-19 group (*p* = 0.004). (Fig. 2).

### Measles virus (Rubeola virus)

**Fig. 3:**
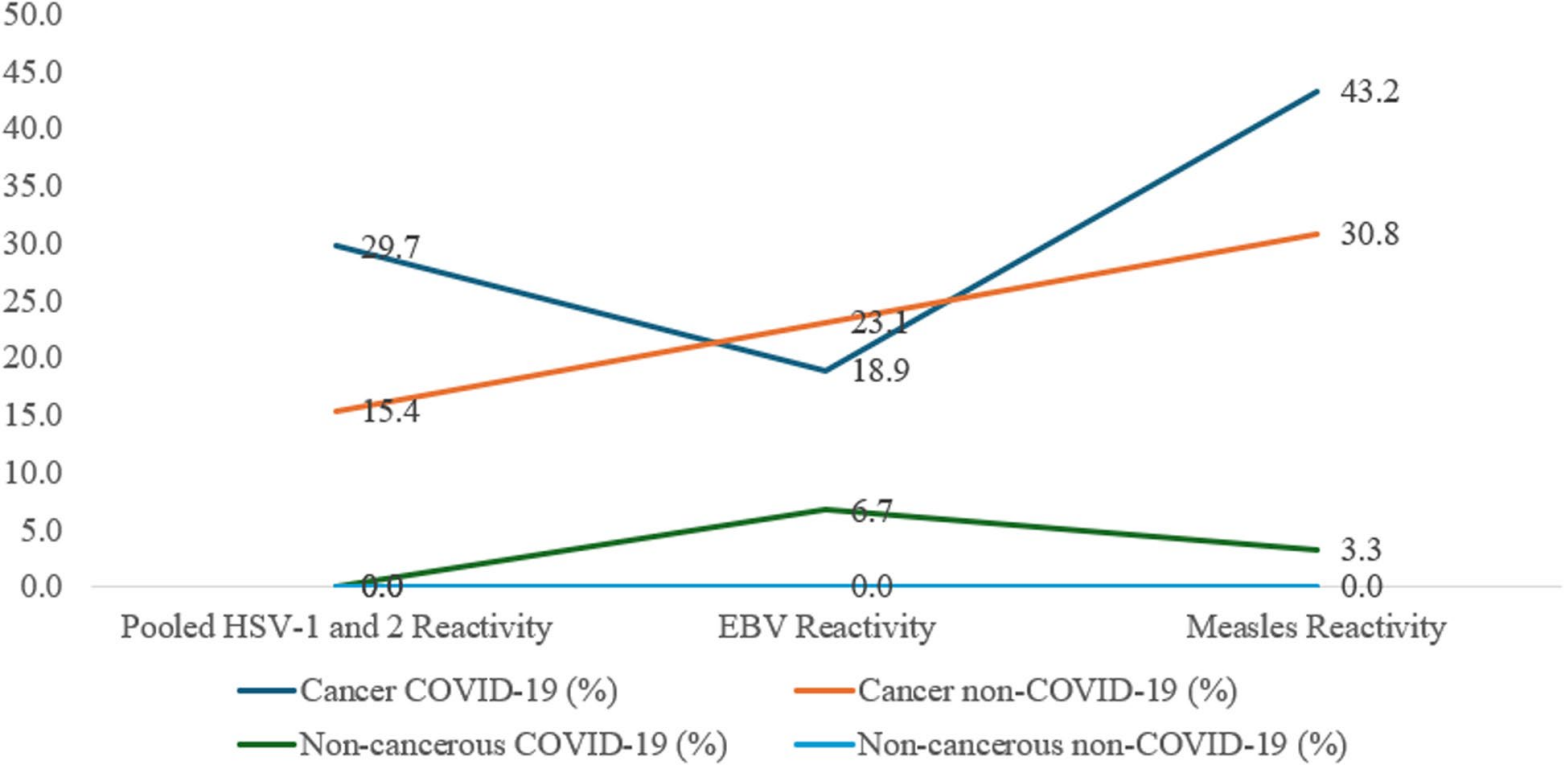
Reactivity, ≥ fourfold increase in IgG in all study groups versus each other’s

About measles virus, transmitted by respiratory droplets, for the IgG, we found that 84.6% and 75.7% of cases were positive in the cancer non-COVID-19 group and cancer COVID-19 group, respectively (Fig. 2). We discovered that 43.2% and 30.8% of cancer patients in the cancer COVID-19 and cancer non-COVID-19 groups, respectively, were reactive for the measles virus, whereas the normal COVID-19 and control groups were 3.3% (only one case) and 0.0% respectively (*p* < 0.001). (Fig. 3). The assay utilized to evaluate MeV IgG titers was quantitative, as opposed to the other serological viral marker kits employed in this research (Table 3).

### Herpesviruses: Pooled HSV1/2, and EBV

When studying certain members from the *Herpesviridae* family, the results showed that 29.7% and 38.5% of the cases were positive in the cancer COVID-19 and cancer non-COVID-19 groups, respectively, and 33.3% of the cases were positive in the normal COVID-19 group, compared to 9.1% in the control group. However, by looking at the pooled HSV1/2 IgM profile specific for herpes simplex viruses 1 and 2, we discovered that the cancer COVID-19 group had 27.0% and the cancer non-COVID-19 group had 7.7% of positive cases, respectively, while the normal COVID-19 group had 51.2% of positive cases, compared to 23.1% in the control group (*p* = 0.002). Moreover, pooled HSV1/2 IgM levels in plasma of COVID-19 patients represents 43.7% while the percentage was15.4% in non-COVID-19 groups (*p* = 0.0007). (Fig. 1).

The current research showed a clear statistical significance by examining reactivation based on active viral infection using immunoglobulin (G) fourfold rise model. By studying HSV1/2 IgG specific to herpes simplex viruses 1 and 2, where we found that 26.0% of cancer cases, cancer COVID-19 and cancer non-COVID-19 groups, tested positive versus 0.0% in non-cancerous one (*p* = 0.007). In addition, 29.7% and 15.4% of cases were positive in both cancer groups, cancer COVID-19 and cancer non-COVID-19 groups, respectively. In comparison, 100.0% of cases with only COVID-19 without cancer were negative cases (*p* = 0.008). Applying the same model for an investigation of the antibody EBNA-1 IgG specific to the Epstein-Barr virus, which belongs to the herpesvirus family, we found that 20.0% of cases with cancer were positive in the malignant groups; in contrast, 4.9% of non-cancerous cases in the normal and control groups (*p* = 0.034). (Fig. 3).

## Discussion

The findings of the current study reveal a significant association between SARS-CoV-2 infection and the reactivation of latent herpesviruses and persistent measles viral infection in cancer patients. Specifically, the data indicate that cancer patients with COVID-19 exhibited a markedly higher incidence of recent HSV1/2 infections and reactivation, as evidenced by a significant increase in pooled HSV1/2 IgM and IgG antibody levels compared to controls. Furthermore, the study found a significant increase in EBV reactivation among cancer patients with COVID-19, highlighting this population's susceptibility to viral reactivations. These results align with previous research suggesting that immunocompromised individuals, such as cancer patients, are at heightened risk for viral reactivations during concurrent infections [23, 38, 39]. The substantial increase in MeV IgG levels in cancer patients, particularly those with COVID-19, highlights the potential for measles virus reactivation in this group, which has critical implications for their clinical management and treatment outcomes [34, 40–42].

Cancer COVID-19 group exhibits significantly lower hemoglobin levels compared to the other groups (*p* = 0.005), which may be attributed to the combined effects of cancer and viral infection leading to anemia. The platelet count is notably higher in cancer non-COVID-19 group, possibly due to cancer-related thrombocytosis, whereas normal COVID-19 group shows a lower platelet count, suggesting a potential impact of the virus on platelet production or survival (*p* = 0.007). The elevated ALT levels in cancer COVID-19 group indicate liver stress, likely exacerbated by the dual burden of cancer and SARS-CoV-2 infection (*p* = 0.004). Similarly, the significantly higher serum creatinine levels in normal COVID-19 group (*p* < 0.001) suggest renal involvement due to the viral infection. Blood urea levels also show significant differences, with normal COVID-19 having the highest levels, indicating possible renal impairment or increased protein catabolism due to the infection (*p* = 0.009). Immunologically, the lower TLC in cancer COVID-19 group compared to normal COVID-19 group (*p* = 0.499) may reflect a more pronounced immune suppression in cancer patients with SARS-CoV-2, as lymphopenia is a common feature in severe COVID-19 cases. The neutrophil count is higher in cancer non-COVID-19 and normal COVID-19 groups, which could be a response to infection and inflammation, although the differences are not statistically significant (*p* = 0.082).

Interestingly, the kit used to assess MeV IgG loads was quantitative, in contrast to the other serological viral marker kits employed in this research. The significant increase in platelet count in MeV IgG positive individuals (*p* = 0.026) suggests a possible response to infection or inflammation. Elevated platelet counts (thrombocytosis) can be a reaction to various conditions, including infections, and might indicate an ongoing inflammatory process. Liver Enzymes: the significant increase in ALT and AST in MeV IgG positive cases (*p*-values, 0.025 and 0.010 respectively) suggests liver involvement or stress, which could be due to the infection or an inflammatory response. Elevated liver enzymes are common in viral infections and can indicate liver inflammation or damage. Studies have shown that infections like SARS-CoV-2 can lead to elevated AST and ALT levels due to direct viral effects on the liver or systemic inflammation. Serum creatinine levels are a marker of kidney function. The significant increase in creatinine in SARS-CoV-2 qRT-PCR positive group (*p* = 0.035) suggests potential kidney involvement or stress. This could be due to the body’s response to the infection or other underlying conditions affecting kidney function. Viral infections can impact kidney function, leading to elevated creatinine levels. Blood urea levels are another indicator of kidney function. The significant increase in blood urea in SARS-CoV-2 qRT-PCR positive individuals further supports the possibility of kidney involvement or stress. Elevated urea levels (*p* = 0.010) can indicate reduced kidney function or increased protein breakdown in the body. Blood urea levels can be elevated in viral infections due to reduced kidney function or increased protein catabolism. This data suggests that certain blood parameters, such as platelet count, AST, ALT, serum creatinine, and blood urea, are significantly affected by the presence of SARS-CoV-2 and MeV IgG antibodies, indicating potential impacts on liver and kidney function [43]. The lack of significant differences in hemoglobin, TLC, lymphocyte, and neutrophil counts suggests that these parameters may be less affected by the viral and antibody status.

COVID-19 can indeed affect the kidneys, primarily through the angiotensin-converting enzyme 2 (ACE2) receptors, which are abundant in kidney cells. The virus uses these receptors to enter cells, potentially leading to kidney damage. This can result in elevated creatinine levels, indicating impaired kidney function. Several mechanisms contribute to this damage such as direct viral infection, causing kidney cell injury. Cytokine storm: An excessive immune response can lead to inflammation and damage. Low oxygen levels: Severe COVID-19 can reduce oxygen levels, affecting kidney function. Managing kidney involvement in COVID-19 includes monitoring kidney function and addressing any acute kidney injury (AKI) that may occur [44].

As medical diagnosis has progressed, nucleic acid detection-based methods have emerged as a dependable and quick method for viral identification. The polymerase chain reaction (PCR) test, which is distinguished by quick detection, high sensitivity, and specificity, is regarded as the backbone among nucleic acid assays for the identification of some viruses. Because it is a straightforward and specific qualitative test, real-time quantitative reverse transcriptase-PCR (qRT-PCR) is therefore highly desirable for the identification of SARS-CoV-2 [45–47]. Furthermore, real-time qRT-PCR provides sufficient sensitivity to greatly aid in the early diagnosis of infection. In the cancer COVID-19 group, it was recognized that 100.0% of the cases were positive and 89.0% of the cases in the normal COVID-19 group were positive using quantitative real-time qRT-PCR, while the cases in the cancer non-COVID-19 and control groups were both negative (*p* = 0.001). This representative estimation of the SARS-CoV-2 loads matched with the clinical feature for each group. However, some cases tested negative in the normal COVID-19 group.

Parmer et al., revealed a group of qRT-PCR negative individuals exhibiting clinical signs and symptoms of acute COVID-19 along with serologic evidence of SARS-CoV-2 infection, which was like that of qRT-PCR confirmed COVID-19 and multifold higher than non-suspects [48]. These qRT-PCR negative patients were about half as likely to obtain treatment as the qRT-PCR confirmed COVID-19 individuals, despite equivalent and matched with disease severity. These results point to a crucial role for clinically diagnosed COVID-19, indicating that in the presence of clinical suspicion and no viable alternative diagnosis, a negative qRT-PCR test should not bar evidence-based COVID-19 treatment, given the persistent threat posed by highly contagious variants and the development of new evidence-based COVID-19 therapies. According to [49], a large number of suspected patients with identical particular computed tomography (CT) images and typical clinical characteristics of COVID-19 were tested negative. It appears that managing COVID-19 is made easier by the combination of real-time qRT-PCR and clinical characteristics. Therefore, a negative result does not rule out COVID-19 infection and should not be the only factor considered when making decisions about patient management or treatment. We looked for COVID-19 viral markers. Evaluating SARS-CoV-2 IgG immunoglobulins, in the cancer COVID-19 group had just 59.5% of positive cases because they are cancer patients and therefor immunocompromised; while in the control group, 100.0% tested positive for SARS-CoV-2 IgG since they were normal and immunocompetent, (*p* = 0.004). SARS-CoV-2 IgG remains in blood and provides long-term immunity [50, 51]. Regarding the result of the IgM and for epidemiological reasons, all the samples were collected during the COVID-19 outbreak, i.e., recent infection with another SARS-CoV-2 variant (different versions) was probable.

Similarly, we found that the MeV IgG was positive in 56.8% and 69.2% of cancer patients in the two cancer groups, respectively, while there was only one instance in each of the normal and control groups (*p* = 0.001). This shows that 5.0% of non-cancer patients and 60.0% of cancer cases were positive (*p* < 0.001), regardless of COVID-19 infection. After a median of 10.4 years, Castiñeiras, Sales [52] in a cross-sectional study discovered that 67.3% of young people who had received two or more doses of the measles-mumps-rubella (MMR) vaccine were seronegative by ELISA. These results imply that current measles susceptibility may be higher than anticipated in situations when immunity is mostly based on vaccine stimuli, and more research is necessary to fully understand this relationship. Age and the amount of time since the last MMR dose were the primary variables linked to declining immunity titers. The MMR vaccine at 12 and 18 months respectively was introduced to routine immunization in Egypt as part of the national vaccination program established in 1984 [53]. This program was part of the government’s efforts to enhance public health and control infectious diseases through vaccination. Furthermore, in our study, 60.0% of cancer cases (n = 50) tested positive (p < 0.001). These cases were associated with older age (mean age: 50.20 ± 16.85, p = 0.041) and lacked MMR vaccination. Instead, they had a history of measles infection, which likely contributed to their durable and persistent IgG titers against the measles virus. Whereas non-cancerous volunteers (n = 40), who were younger (mean age: 23.40 ± 5.60, p = 0.041) and MMR-vaccinated, included only two cases that tested positive for measles IgG. According to Al Balakosy, Alfishawy [42] 68% of COVID-19 patients tested positive for measles IgG. Furthermore, patients with positive measles IgG were older than those with negative measles IgG. This was attributed to the fact that monovalent measles vaccine was introduced in Egypt in 1977, and measles vaccination coverage increased from 50 to 90% from 1980 to 1999 [54, 55]. Therefore, the older population was likely to have acquired wild measles infection, which recovery is associated with sustained levels of neutralizing antibody and life-long protective immunity. MeV RNA persists in lymphoid tissue and the immune system remains activated for many months [55]. Furthermore, similarities to SARS-CoV-2 structure plays a crucial role in cross-reaction in relation to other persistent respiratory viruses such as measles. Sidiq, Sabir [56] confirmed by computational homology modeling that that MMR vaccine could provide a broad neutralizing antibody against numbers of diseases, including COVID-19, based on the 30 amino acid sequence homologies between the SARS-CoV-2 Spike (S) glycoprotein (PDB: 6VSB) as antigenic epitopes of both the MeV fusion (F1) glycoprotein (PDB: 5YXW_B). Consequently, compared to adults and the elderly MMR-vaccinated children were less susceptible to COVID-19 due to humoral immunity conferred through the MMR vaccination [57]. Moreover, increased IgG titer in SARS-CoV-2 infected patients was correlated with disease burden, the secondary immune response might be responsible for increased antibody production knowing that antigen homology with SARS-CoV-2 [56]. Even the seroprevalence of measles has not been thoroughly studied in cancer patients. A study conducted by Marquis, Logue [58] found that approximately 25% of their study cohort did not have protective measles antibodies, indicating an increased risk for cancer patients to contract the measles virus. There is an evolutionary relationship between measles virus and Coronavirus. It was believed that measles vaccination could bolster the immune response against COVID-19 [59]. However, a study by Salamony, Shamikh [60] revealed that MeV antibodies may not provide protection against SARS-CoV-2 infection, instead they even may lead to worse patient outcomes and increased ICU admissions. Furthermore, the research indicated that patients with severe COVID-19 exhibited higher levels of MeV antibodies [60].

Herpes simplex viruses (HSVs) are classified into oral herpes (HSV-1) and genital herpes (HSV-2) [61]. Shanshal and Ahmed [25] reported that 35% of the study cohort (eighty mild to moderate COVID-19 patients) exhibited at least one HSV infection. It is also well established that reactivation of the HSV occurs in non-immunocompromised patients undergoing prolonged mechanical ventilation, and this condition is linked to a heightened risk of mortality. Given that COVID-related acute respiratory distress syndrome (ARDS) often requires mechanical ventilation, there is an elevated risk of HSV reactivation in these patients [62, 63].

According to the pooled HSV1/2 IgM profile specific for herpes simplex viruses 1 and 2, recent infection was detected in 33.3% of COVID-19-diseased patients in the normal COVID-19 group, compared to 9.1% in the control group. In the malignant groups, 29.7% and 38.5% of cases tested positive, respectively. Nevertheless, the cancer COVID-19 group had 27.0% and the cancer non-COVID-19 group had 7.7% of positive cases, respectively, the normal COVID-19 group had 51.2% of positive cases, compared to 23.1% in the control group (*p* = 0.002). Additionally, the percentage of pooled HSV1/2 IgM levels in plasma from COVID-19 patients is 43.7%, compared to 15.4% in non-COVID-19 groups (*p* = 0.007).

Moreover, 26.0% of cases in the malignant groups tested positive for HSV1/2 IgG specific to herpes simplex viruses 1 and 2, compared to 0.0% in the non-cancerous group (*p* = 0.007). Furthermore, the percentage of positive cases in the cancer groups were 29.7% and 15.4%, respectively. On the other hand, 100.0% of cases with just COVID-19 and no cancer were considered negative (*p* = 0.008). Using the same model, we examined the antibody EBNA-1 IgG specific to EBV. We discovered that, in the first two groups, 20.0% of cancer cases tested positive, while in normal COVID-19 and control groups, 95.1% of non-cancerous cases tested negative (*p* = 0.034).

Our study highlights the interplay between SARS-CoV-2, herpesviruses (HHV), and measles virus (MeV) in cancer patients, revealing a significant risk of viral co-infections and reactivations in this immunocompromised population. The observed reactivation of HHV and MeV in SARS-CoV-2-infected cancer patients may be driven by several interconnected mechanisms, which warrant further exploration.

First, immune dysregulation induced by SARS-CoV-2 infection likely plays a central role in viral reactivation. SARS-CoV-2 is known to cause profound alterations in the host immune response, including lymphopenia, cytokine storm, and impaired T-cell function [64, 65]. These immune perturbations may create a permissive environment for the reactivation of latent viruses such as HHV and MeV, which are typically controlled by robust cellular immunity [66, 67]. In cancer patients, who already exhibit baseline immune suppression due to their underlying disease and treatments, these effects may be further exacerbated, increasing the risk of viral reactivation.

Second, direct viral interactions between SARS-CoV-2 and other viruses may contribute to reactivation [68]. For example, SARS-CoV-2 has been shown to modulate host cell pathways, such as the interferon response, which could inadvertently promote the replication of co-infecting viruses [69]. Additionally, the inflammatory ambiance created by SARS-CoV-2 infection may upregulate cellular factors that facilitate the replication of HHV and MeV [70].

Third, cancer-related factors may further amplify the risk of viral reactivation. Cancer itself can impair immune surveillance, creating a favorable environment for latent viruses to re-emerge [38, 68, 71]. In our study, the higher rates of HHV and MeV reactivation in SARS-CoV-2-infected cancer patients compared to non-cancer individuals support the conception that cancer-related immune suppression synergizes with SARS-CoV-2-induced immune dysregulation to drive viral reactivation.

Finally, host genetic and epigenetic factors may also influence the likelihood of viral reactivation. Variations in immune-related genes or epigenetic modifications induced by SARS-CoV-2 infection could alter the host’s ability to control latent viruses [72]. While our study did not explore these factors, they represent an important area for future research.

In conclusion, the reactivation of HHV and MeV in SARS-CoV-2-infected cancer patients is likely mediated by a combination of immune dysregulation, direct viral interactions, cancer-related immune suppression, and host factors. These findings underscore the importance of monitoring for viral co-infections and reactivations in immunocompromised populations, particularly during the ongoing COVID-19 pandemic. Future studies should investigate these mechanisms in greater detail to inform targeted therapeutic strategies.

## Conclusions

In conclusion, this research broadens our knowledge on cancer and COVID-19 features such as viral co-infections and reactivation suggesting a potential role in MeV, HSV1/2 (HHV1-HHV2) and EBV reactivation. Based on our experience with the current evaluation, additional research on virus reactivation in cancer and/or COVID-19 patients is necessary. Regarding the high clearance rate of measles antibody seropositivity among young population who have previously received two doses of a MMR vaccine, we recommend an additional booster dose.

Overall, the data underscores the compounded impact of SARS-CoV-2 on cancer patients, affecting various hematological parameters and indicating significant stress on the liver and kidneys. The immune response, as evidenced by lymphocyte and neutrophil counts, varies across the groups, reflecting the complex interplay between cancer, viral infection, and the body’s immune system.

## Methods

### Patients

We conducted a prospective observational study on newly diagnosed cancer patients infected with SARS-CoV-2. The study recruited all eligible patients between September 2020 and September 2022 at the NCI, Cairo University. The study began by recruiting newly diagnosed cancer patients infected with SARS-CoV-2 (n = 37), who were then followed to assess clinical outcomes. Then apparently normal individuals infected with SARS-CoV-2 (*n* = 82) and treatment naïve cancer patient non-infected with SARS-CoV-2 (*n* = 13) as control were recruited. In addition, a normal group non-infected with the virus is included (*n* = 15). Therefore, the study was conducted on 4 groups (*n* = 147).

### Selection of control groups

The control groups in this study were selected based on specific inclusion criteria. The Noncancerous/COVID group (*n* = 82) consisted of apparently normal individuals; however infected with SARS-CoV-2, confirmed by qRT-PCR. The Control group (*n* = 15) included completely apparently healthy individuals without SARS-CoV-2 infection, confirmed by qRT-PCR and serological testing. Due to the challenges of recruiting a sufficient number of healthy individuals during the COVID-19 pandemic, we tried our best to have age and gender compatible in the standard set.

### Molecular detection of SARS-CoV-2

- a. Nucleic Acid Extraction

Nasopharyngeal swab (NPS) specimens were collected on a viral transfer medium (VTM). Viral RNA of SARS-CoV-2 for all cancer and normal cases was extracted from NPS samples using QIAamp® MinElute Virus Spin Kit, Cat# 57,704, (Qiagen, USA) according to the manufacturer's instructions.

- b. Detection of SARS-CoV-2 RNA

The real-rime qRT-PCR was performed to measure the viral titer of SARS-CoV-2 using TaqPath™ COVID-19 CE-IVD One-Step RT-PCR kit (Cat# A48067) Thermofisher™, USA. According to the manufacturer's protocol, samples were run on the 7500 Fast Real-Time PCR System instrument (Applied Biosystems, Cat# 2750142R) using the 40-cycle RT-PCR protocol. Data were analyzed using the 7500 Software version 2.0.5 (Applied Biosystems Co.) at NCI. A positive qRT-PCR test converts a questionable case into a confirmed COVID-19 cancer patient, which is defined as a person who has laboratory confirmation of SARS-CoV-2 infection, regardless of clinical signs and symptoms. Whereas in non-cancerous cases a positive COVID-19 case show respiratory symptoms such as fever, cough, and respiratory difficulties [51].

### Serological assay

- a. Enzyme-linked immunosorbent assay (ELISA)

After collection of venous blood samples from all studied groups using sterile vacutainer tubes with anticoagulant, plasma samples were separated by centrifugation at 600 XG for 3 min at 20 °C. Samples were carefully aliquoted into labeled cryovials and immediately stored at -80 °C until analysis. The enzyme-linked immunosorbent assay (ELISA) is the methodology employed to measure the anti-bodies of SARS-CoV-2 IgG and IgM, pooled HSV-1 and HSV-2 IgG and IgM, EBNA-1 IgG, and MeV IgG in the plasma of patients and controls samples using TECAN’s Sunrise™ absorbance microplate reader and commercially available ELISA kits, according to the instructions of the manufacturer. Microplates (96-well) were purchased from leading medical manufacturer companies for laboratory diagnostics in human plasma, such as EUROIMMUN for the detection of SARS-CoV-2 IgG and IgM, as well as detection of antibodies against pooled Herpes Simplex Virus types 1 and 2 (HSV1/2), and DRG for the detection of IgG antibodies against Epstein-Barr Nuclear Antigen 1 (EBNA-1).

- b. Calculations of 4-parameter method

After determination of the measles virus IgG titer in plasma samples, using the RIDASCREEN® Measles virus IgG (K5421) kit; a 4-parameter calculation method outlined in the enclosed data sheet of the manufacturer was employed. The key equation involves compensation: EXP(C-LN((D-A)/(Sample*SC/S1-A)-1)/B). The four parameters’ values were: C = 8.2811, D = 3.7627, A = − 0.0607, and B = 0.7304. Eventually our final equation was: EXP (8.2811-LN((3.7627-(-0.0607))/(Sample*0.897/S1-(-0.0607))-1)/0.7304).

- c. Reactivity measurement using fourfold criterion

According to Aruta, Lari [73] ELISA is one of the most popular techniques for assessing immune response reported as a fourfold increase relative to baseline. Evidence of herpesviruses reactivation is defined as a fourfold increase in IgG titer [74, 75]. Moreover, measuring recent infection in certain situations may be aided by a fourfold increase in MeV IgG titers between acute and convalescent samples [76].

### Statistical methods

The data was analyzed using SPSS version 23. Quantitative data were given as mean and standard deviation (or median, minimum and maximum for non-parametric data). Qualitative data were provided as counts and percentages. Non-parametric data was analyzed using the Kruskal–Wallis H test. Mann Whitney and the Independent sample t-test was used to compare non-parametric quantitative data from two independent groups. The Chi-Square and Fisher exact tests were employed to compare qualitative data between groups. The Spearman's correlation test was used to compare the correlation between continuous variables. The *p-*values less than or equal to 0.05 were considered statistically significant.

## Data Availability

No datasets were generated or analysed during the current study.

## Abbreviations

MeV: Measles virus
HHV: Human herpesvirus
ELISA: Enzyme-linked immunosorbent assay
qRT-PCR: The quantitative reverse transcription polymerase chain reaction
HSV1/2: Herpes simplex viruses 1 and 2
EBV: Epstein-Barr virus
IgG: Immunoglobulin G
IgM: Immunoglobulin M
RNA: Ribonucleic acid
IRP: Institutional review board
COVID-19: Corona virus disease 2019
SARS-CoV-2: Severe acute respiratory syndrome corona virus-2
EBNA-1: Epstein-Barr Nuclear Antigen-1
